# The Existence and Stability Mechanism of Bulk Nanobubbles: A Review

**DOI:** 10.3390/nano15040314

**Published:** 2025-02-18

**Authors:** Changsheng Chen, Yawen Gao, Xianren Zhang

**Affiliations:** 1 New Cornerstone Science Laboratory, Center for Combustion Energy, Key Laboratory for Thermal Science and Power Engineering of Ministry of Education, Department of Energy and Power Engineering, Tsinghua University, Beijing 100084, China; 2 State Key Laboratory of Organic–Inorganic Composites, Beijing University of Chemical Technology, Beijing 100029, China

**Keywords:** bulk nanobubbles, stability, interfacial charge, compressed monolayer, interfacial thermal fluctuations

## Abstract

Since they were shown to be a potential phenomenon through experimentation, bulk nanobubbles (BNBs) have been a long-standing controversy. The controversy mainly originates from the fact that their stability cannot be well explained by the established theories. Although nanobubbles have been applied in many fields, the controversial stability issue has been a hanging “cloud” looming over the nanobubble research. This review focuses on why the stability of nanobubbles cannot be depicted by the current theories from thermodynamics and dynamics perspectives. Moreover, a number of current models pertaining to bulk nanobubble stability are compiled. It is anticipated that this review will give readers a better grasp of the current state of bulk nanobubble research and provide some insight for further studies in this area.

## 1. Introduction

Nanobubbles are fine bubbles that have at least one dimension in the range of 1–1000 nm [1,2,3,4,5,6,7,8]. According to their existence form, they can be divided into two categories: one is the surface nanobubbles (SNBs) that are attached to the immersed solid interface, and the other is the bulk nanobubbles (BNBs) that are suspended in the solution. Nanobubbles have attracted the interest of researchers because of their wide applications in various areas, such as water treatment [9,10,11,12], flotation [13,14,15,16,17], cleaning [18,19,20,21], and chemical regulation [22,23]. In recent years, the number of reports regarding nanobubbles has increased rapidly. This trend has been particularly evident since the International Conference on Nanobubbles held in Suzhou, China, in 2018, with an annual growth rate exceeding 10%. This clearly indicates that nanobubbles are emerging as a potential technique with various applications as well as a popular field of study [6,7,8].

Currently, the most fundamental scientific problem about nanobubbles is their -unexpected stability [2,3,4,5,6,7]. This is also the most controversial issue that has not been completely solved. For SNBs, the contact line pinning-supersaturation mechanism proposed by Zhang et al. and Lohse et al. [1,24,25,26] can basically explain the stability of SNBs in most cases. Regarding the stability of BNBs, although different mechanisms have been proposed in the past 20 years, such as the interface charge model [27,28], the contaminant model [29,30], the locally supersaturated cluster model [31,32], etc., a consensus has not yet been reached.

The reason the stability of bulk nanobubbles cannot be explained by classical theory is that the small size and high curvature cause the gas pressure inside the bubbles to be extremely high. According to the classical Laplace equation, Δ*p* = 2*γ*/*R*, and the Epstein–Plesset (E-P) equation, the survival time of bubbles with a radius of 100 nm is estimated to be ~0.02 s [33], which is completely inconsistent with the experimentally observed nanobubbles that can survive for several hours or even tens of days [2,3,4,5]. This is known as the Laplace catastrophe of nanobubbles [2,34]. It is also the main reason some researchers oppose the long-term survival of BNBs [2,35,36,37,38].

Although the stability mechanisms of BNBs remain controversial, the application of micro/nanobubbles has advanced significantly, far ahead of the fundamental theoretical research [9,10,11,12,13,14,15,16,17,18,19,20,21,22,23]. Therefore, it is urgent to make breakthroughs in the stability study of BNBs from the basic research and remove the “dark cloud” of nanobubbles. Based on the current research progress, this review briefly overviews the stability of BNBs from the following three aspects: (1) The existence and controversy of BNBs; (2) Several proposed stability mechanisms of BNBs; (3) Summary and outlook. It should be stated that the review primarily focuses on the theoretical aspects of the stability of BNBs, as it does not cover all related studies, such as experiments and applications.

## 2. The Existence and Controversies of Bulk Nanobubbles

### 2.1. The Uncertainty of Experimental Observations

Despite the fact that BNBs were initially discovered through experimental studies, one disadvantage of BNBs studies is the lack of direct evidence that the reported nano-objects are really nanobubbles, due to the limitations of experimental techniques. Currently, the main instruments for experimentally observing BNBs are dynamic light scattering (DLS) and nanoparticle tracking analysis (NTA). Other measurement techniques, such as digital holographic microscopy, resonant mass measurement (RMM), and electron microscopy (EM), are not widely used at present due to the demanding requirements on the measurement sample and the in situ impact on the sample.

(1) DLS: The autocorrelation function of scattered light intensity decays serves as the foundation for dynamic light scattering. For the size distribution, the obtained DLS data are merely the mean value derived from the autocorrelation function fit. Lack of precise information will lead to more confusion in subsequent data analysis because BNBs suspensions frequently contain bubbles of varying sizes, and the gas/bubble interface has a very high refractive index. The inability of DLS to separate gas nanobubbles from particles or droplets based solely on chemical information is another problem [35,36,37].
(2) NTA: NTA technique can capture the movement of each scattering object in the solution with dark field microscopy and analyzes their trajectories to derive their sizes, respectively, according to the Stokes–Einstein relationship of Brownian motion: $D_{d}=k_{B}T/(3\pi \eta d)$, where *d* is the motion diameter of the observed entity and *D_d_* is the diffusion coefficient. Zhou et al. recently found that the nanobubble diameter *d* counted by the Stokes–Einstein equation is slightly larger than the actual value relative to nanoparticles by molecular dynamics simulation (MD) studies [39]. Favorably, compared to DLS, the NTA technique is much more accurate and reliable for size determination, with a typical reliable particle size around 30–1000 nm. However, the disadvantage of this technique is also that they cannot distinguish gas nanobubbles from nanoparticles or nanodroplets.

The aforementioned deficiencies may be the main reason for some controversies: Are the observed nano entities in some literature reports nanobubbles, nanodroplets, or nanoparticles? [35,36,37,38]. This ongoing controversy is not only due to the uncertainty of experimental observations but also the conflict between the stability of BNBs and classical theories.

### 2.2. In Conflict with the Classical Theory

Aqueous solutions containing BNBs in most applications and experimental observations are open to the atmosphere. Based on these circumstances, we can give a simple theoretical analysis below to show that BNBs cannot be stable theoretically.

In aqueous solution with dissolved gases, the gas inside a nanobubble is mainly composed of dissolved air (gas) and a small amount of water vapor. Assuming that the chemical potential of gas in the solution is constant (μVT ensemble), the free energy change during the expansion or contraction of a spherical nanobubble with volume *V* and surface area *A* can be expressed in terms of the grand potential *Ω*, whose differential form reads

(1) $$d\left(\Delta \Omega \right)=-\Delta \mathrm{pdV}+\mathrm{\gamma dA}$$

where $∆p=p_{\mathrm{in}}-p_{0}$ is the pressure difference between the interior and exterior of the nanobubble, γ is the interfacial tension of the gas–liquid interface. In the μVT ensemble (isothermal, isochemical potential system), the grand potential *Ω* can be used to express the free energy of the system. This is analogous to the fact that the free energy of the canonical ensemble (NVT) and the isothermal isobaric ensemble (NPT) can be expressed in terms of the Helmholtz free energy *F* and the Gibbs free energy *G*. Here, we assume that the chemical potential of the gas molecules in the solution is always constant (μVT ensemble). Henry’s law predicts that the pressure inside a bubble is $p_{\mathrm{in}}=p_{g}+p_{v}=Hc_{\infty }+p_{v}=H\left(1+\xi \right)c_{g}^{\mathrm{sat}}+p_{v}$, where *H* is Henry’s coefficient, $c_{\infty }$ is the concentration of gas in the aqueous solutions, $c_{g}^{\mathrm{sat}}$ is the saturated concentration of dissolved gas under the environmental pressure $p_{0}$ ($p_{0}=p_{g}^{\mathrm{sat}}+p_{v}$), the gas supersaturation is defined as $\xi =c_{\infty }/c_{g}^{\mathrm{sat}}-1$. For saturated gas solubility, Henry’s law requires $p_{g}^{\mathrm{sat}}=Hc_{g}^{\mathrm{sat}}$. Thus, $∆p=p_{\mathrm{in}}-p_{0}=p_{g}^{\mathrm{sat}}\xi \approx p_{0}\xi$, where we have assumed $p_{g}^{\mathrm{sat}}\gg p_{v}$, $p_{g}^{\mathrm{sat}}\approx p_{0}$.

Taking the surface tension of a nanobubble *γ* = 0.072 N/m, the excess grand potential Δ*Ω* varying with bubble radius *R* can be determined from Equation (1) by integration. When the surface tension remains unchanged, there is only one equilibrium state, which can be determined from Equation (2)

(2) $$\partial \left(\Delta \Omega \right)/\partial R=0$$

**Figure 1:**
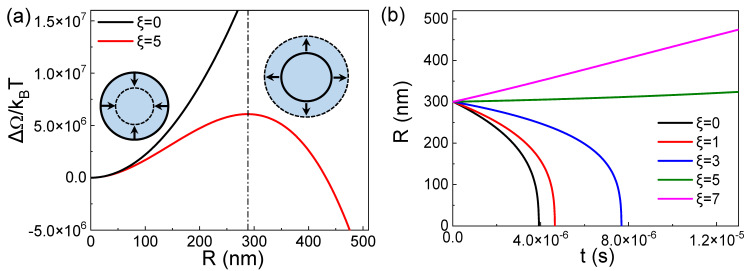
(**a**) Evolution curve of nanobubble free energy Δ*Ω* with bubble radius *R* at *ξ* = 5. (**b**) Dynamic curves of bubble radius *R* with time *t* at different supersaturation *ξ*.

The equilibrium state actually corresponds to the maximum value of Δ*Ω*(*R*), as shown in Figure 1a. The figure shows that when the bubble is in equilibrium (the maximum point corresponding to the dotted line in the figure), a slight perturbation causes the bubble to either dissolve and disappear or grow indefinitely. The free energy continues to decrease with decreasing radius *R* on the left side of the dotted line or continues to decrease with increasing *R* on the right side of the dotted line. Therefore, from the perspective of classical thermodynamics, BNBs cannot exist stably. This means that regardless of the value of the surface tension, the BNBs in the solution without adsorption at the gas–liquid interface are always in a thermodynamically unstable state.

In the following, we shall analyze the stability of BNBs from the viewpoint of bubble dissolution dynamics. For an isolated nanobubble of radius *R*, its dissolution dynamics can be described by the E–P equation [33],

(3) $$\frac{\mathrm{dR}}{\mathrm{dt}}=\frac{D\left(c_{\infty }-c\left(t\right)\right)}{\rho_{g}}\cdot \left(\frac{1}{R}+\frac{1}{\sqrt{\pi \mathrm{Dt}}}\right)$$

where *D* is the diffusion coefficient of gas, $c_{\infty }$ is the concentration of gas in the bulk solution far from the bubble, and $\rho_{g}$ is the density of gas inside the bubble. The gas concentration $c\left(t\right)$ at the bubble surface can be given by Henry’s law

(4) $$c\left(t\right)=\frac{1}{H}p_{\mathrm{in}}\left(t\right)\approx \frac{c_{g}^{\mathrm{sat}}}{p_{0}}\left(p_{0}+\frac{2\gamma }{R}\right)=\left(1+\frac{2\gamma }{Rp_{0}}\right)c_{g}^{\mathrm{sat}}$$

where $c_{g}^{\mathrm{sat}}$ is the saturated concentration of the gas under the environmental pressure $p_{0}$. By substituting $c_{\infty }=\left(1+\xi \right)c_{g}^{\mathrm{sat}}$ and Equation (4) into Equation (3), we can obtain

(5) $$\frac{\mathrm{dR}}{\mathrm{dt}}=\frac{Dc_{g}^{\mathrm{sat}}\left(\xi -\frac{2\gamma }{Rp_{0}}\right)}{\rho_{g}}\cdot \left(\frac{1}{R}+\frac{1}{\sqrt{\pi \mathrm{Dt}}}\right)$$

According to Equation (5), the dynamics evolution curves of the BNBs radius *R* with time under different gas saturations can be obtained. The result is shown in Figure 1b. The result shows that the bubbles will expand in a high gas supersaturated environment and dissolve in a low gas saturation, and the dissolution time is in the order of μs. This result is consistent with the thermodynamic result in Figure 1a.

In summary, classical thermodynamic and dynamic theories all predict that BNBs cannot stably exist for a long period of time. Indeed, there have been continuous doubts about the existence of BNBs. Experimental reports suggest that the observed nanobubbles may be nano-droplets or nano-particles [35,36,37,38], leading to possible explanations for the existence of nano-droplets or contamination instead of nanobubbles [38]. However, considering the fact that many applications of nanobubbles are not easily explained by nano-droplets or nano contaminants, researchers have endeavored to provide evidence for the possible existence of BNBs. For example, some degassing and pressure regulation experiments [40,41,42,43] may indicate that the observed nano-entities are most likely BNBs, at least partially.

## 3. Stabilization Mechanism of Bulk Nanobubbles

Based on the fact that BNBs are very likely to exist, several theoretical models have been developed to account for the stability of BNBs in aqueous solution. The models proposed so far include surface charge potential model, impurity model, diffusion shield model, dynamic equilibrium model, etc. Although these models mostly remain uncertain, they generally provide good inspiration for future study.

### 3.1. Interface Charge Model (Zeta Potential Model)

**Figure 2:**
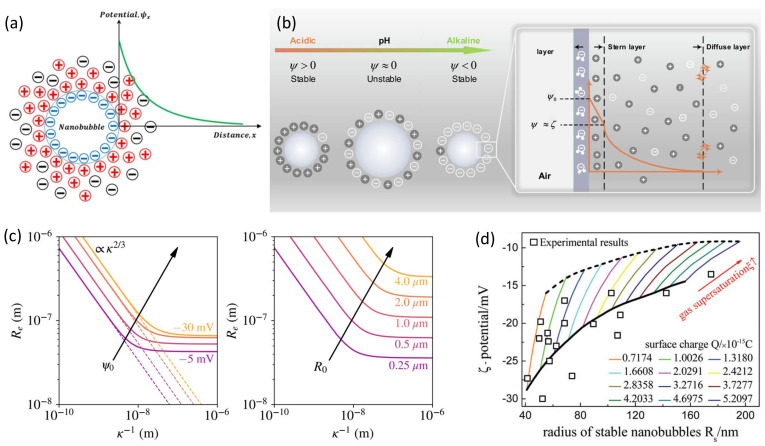
(**a**) Schematic of the surface charge model of BNBs [45]. (**b**) Schematic of the charged nanobubbles under various pH and diffused electrical double layers formed around a negatively charged surface [47]. (**c**) Stable equilibrium radii *R*_e_ of BNBs under the range of Debye lengths of 1 Å < κ^−1^ < 1 μm, for *ξ* = 0 [28]. (**d**) Relationship between bubble radius and Zeta potential under different amounts of charges adsorbed and gas supersaturation; here the square symbols represent the experimental data [27].

The interface charge model assumes that there is a net charge layer adsorbed on the gas–liquid interface of the BNBs [27,28], as shown in Figure 2a, and the charge is usually negative. The mutual repulsion between charges forms an equivalent electrostatic pressure $p_{Q}$, which can be described by

(6) $$p_{Q}=\frac{\sigma^{2}}{2\varepsilon_{r}\varepsilon_{0}}$$

where *σ* is the surface charge density on the bubble, $\varepsilon_{0}$ is the permittivity of vacuum, and $\varepsilon_{r}$ is the relative permittivity of water. $p_{Q}$ acts opposite to the Laplace pressure Δ*p*. It is equivalent to effectively reducing the surface tension, thereby reducing the Laplace pressure Δ*p* = 2*γ*/*R* caused by the surface tension. Thus, the internal pressure of the bubbles can be reduced to relieve the Laplace catastrophe.

In 2007, the surface charge model of BNBs was proposed and discussed by Jin et al. [44], and numerous experimental studies have demonstrated that both solution pH and the concentration and type of salt solution exert significant impacts on the stability of BNBs [45,46,47,48,49,50]. Based on the hypothesis that the enrichment of interfacial charge will lead to the formation of an electrical double layer at the gas–liquid interface of the bubble (forming Zeta potential), in 2020, Ohl et al. [27] and Zhang et al. [28], respectively, gave specific models for calculating *σ*(*R*) by solving the Poisson–Boltzmann equation under special conditions,

(7) $$\sigma (R)=\frac{2\varepsilon_{r}\varepsilon_{0}\kappa k_{B}T}{e}\sinh\left(\frac{e\psi }{2k_{B}T}\right)f(R)$$

Here, the Zeta potential *ψ* is the electric potential at the distance from the bubble that delineates mobile bulk liquid from an immobile liquid at the double layer, and *f*(*R*) is a correction term that accounts for the curvature of the double layer. They obtained some results by calculating with the theory, which are in good agreement with experimental trends [45,46,47,48,49,50,51,52,53,54,55,56,57], as shown in Figure 2c,d.

The interfacial charge model can partially explain experimental results, as well as some pH and salt solution effects on the nanobubble stability in the experimental trends. It can also explain that BNBs will not coalesce due to mutual repulsion. But the model still has the following disadvantages:

(1) Stabilization of BNBs without gas supersaturated solution environments (*ξ* = 0) requires the Zeta potential in excess of 500 mV, which does not correspond to the experimentally observed −20 to −50 mV.
(2) According to this model, all sizes of stable nanobubbles can be obtained by adjusting the interfacial charge density. This is inconsistent with the experimental observation that the diameter of nanobubbles is always in the range of 100–400 nm regardless of how the dissolved ion concentration is adjusted. This suggests that some important factors that determine the size of BNBs are not captured by the model.
(3) Pure water without any ions also exhibits certain concentrations of BNBs, although their concentration is lower than in solutions containing ions or surfactants. This indicates that in at least some cases, stable BNBs do not require ions in the solution.
(4) The interfacial charge model is currently not fully confirmed by a powerful tool at the microscopic level, the molecular dynamics simulation (MD). MD under classical force fields does not seem to confirm the existence of interfacial charge [58,59]. However, there has been a new development of deep potential molecular dynamics simulations (DPMD) that confirms that there can be net charge enrichment at the gas–liquid interface in some cases [60], which is a significant progress of MD in the field of nanobubble research.

### 3.2. Mechanisms of Interfacial Adsorption of Contaminants

Similarly to the interfacial charge model, a number of experiments have provided substantial evidence that adding amphiphilic ethanol or surfactants could stabilize a large number of BNBs [61,62,63,64,65,66,67,68,69]. This fully illustrates that these surface-active substances can promote the stability of BNBs. Compared with the surface charge model, the model has been verified by some careful experiments [30]; organic contaminants may be an important source of stabilizing BNBs. Even in pure water, due to trace contaminants (<50 ppm), a certain concentration of BNBs can still be stabilized. The theory can explain nanobubbles in pure water to a certain extent [70]. Although their explanations are reasonable, the inadequacy of the theory is exposed in quantitative calculations. According to the classical Langmuir adsorption equation,

(8) $$\gamma =\gamma_{w}-\bar{R}T\Gamma_{\infty }\ln(1+c_{\mathrm{am}}/b)$$

where $\bar{R}$ is the gas constant, *Γ*_∞_ is the maximal surface coverage of the surfactant, *b* is the Langmuir constant of the surfactant. Even when the gas–liquid interface is completely covered with a surfactant like ethanol, the interfacial tension is only reduced to the interfacial tension of ethanol, 0.022 N/m, rather than 0. This implies that the stabilization of BNBs still requires a supersaturation of the gas (*ξ*~4), which is inconsistent with the practical gas supersaturation *ξ* close to 0. In addition, the model cannot interpret that the experimental observed BNBs size is always in the range of 100–400 nm, instead of covering the entire size. Correspondingly, the contaminant model does not explain the trend of charge effects on stability of BNBs. Consequently, the model is likewise not well accepted.

### 3.3. Cluster Diffusion Shield Mechanism

Experimental studies suggest that BNBs with ~100 nm are likely composed of gas clusters of 1–2 nm in size [32]. These clusters can disperse or aggregate under some conditions. There may be a high supersaturation of gas around these small clusters, and the diffusion shield can be formed between neighboring bubbles, hindering their dissolution. Such a model has been confirmed to be able to exist in some small-scale MD simulations [31,60,71,72,73,74]. However, there is still an unsolved question of how this local supersaturation can be maintained for a long time without diffusional dissolution into the surrounding solution where there is no gas supersaturation (*ξ*~0). Similarly, why the observed sizes of nanobubbles in experiments are always in the range of 100–400 nm, and how the surfactants and charges influence their stability, cannot be explained by this model either.

### 3.4. Thermal Fluctuation Model of Gas–Liquid Interface

**Figure 3:**
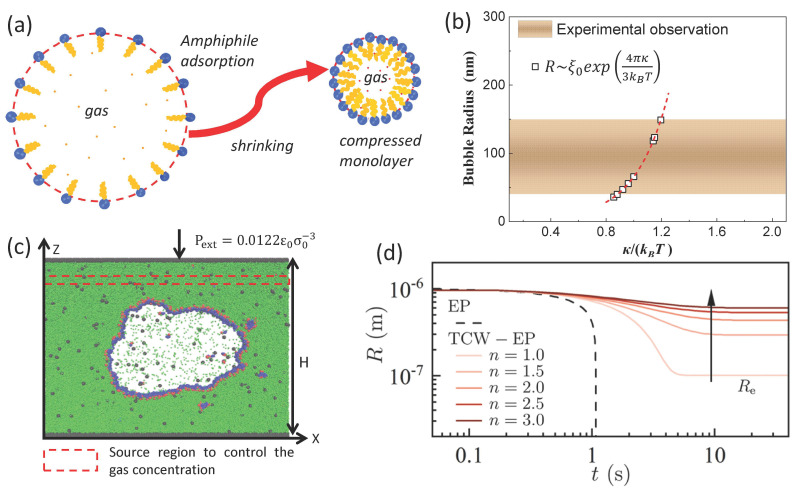
(**a**) Schematic drawing of the formation of a compressed amphiphilic monolayer when a microbubble shrinks. (**b**) Comparison of predicted nanobubble radius with the range of radii of stable nanobubbles reported experimentally. (**c**) The final MD configuration of a bulk gas nanobubble covered by surfactants [76]. (**d**) Evolution of nanobubble *R* from E-P and thermal capillary waves (TCW)-EP models [75].

Recently, Chen et al. [75] interpreted the long-term life of BNBs from a new perspective, interfacial thermal fluctuation (Figure 3d). This model greatly expanded the horizons of our understanding of nanobubbles and is a novel perspective. In addition, Chen and Zhang et al. [70,76] proposed a nanobubble interfacial compression monolayer model (surface tension of gas–liquid interface that can be reduced to zero, *γ*~0), as shown in Figure 3a,c to explain the stability of BNBs under no gas supersaturation (*ξ*~0). The thermal fluctuation effect of the interfacial layer is also used to explain the typical nanobubble size of 100–400 nm observed experimentally (Figure 3b). The compression monolayer is mainly formed by the contraction process of microbubbles [77,78]. And the model has been confirmed by experiment [79].

However, the detailed microstructure interpretation of the interfacial monolayer and the specific verification of its thermal fluctuation effect still need further detailed investigation. In addition, nanobubbles still exist under freezing conditions, and how the influence of ions in dissolution on nanobubbles is considered also needs to be further clarified.

### 3.5. Other Models

In addition to the above-mentioned models, Yasui et al. proposed a dynamic-equilibrium model to explain the stability of BNBs [80,81]. They argued that hydrophobic substances in the solution adsorbed on one side of the nanobubbles, capturing a constant flow of gas for the bubbles, supplementing the gas diffusing out from the uncovered side of the bubbles, and realizing the dynamic equilibrium of the BNBs. However, the model failed to explain the problem of the source of energy needed to maintain the continuous movement of gases.

Furthermore, Wang et al. [29] proposed a curvature-dependent interfacial tension model induced by the interface membrane, *γ*(*R*), which can well explain the stability of BNBs, inhibiting the Ostwald ripening behavior. In this model, the thermodynamic and dynamic equilibrium conditions are met at the equilibrium radius *R*_e_, i.e., $\frac{\partial \Delta G}{\partial R}=0$, $\frac{\partial^{2}\Delta G}{\partial R^{2}}>0$, $\frac{dR}{dt}=0$, from the perspective of thermodynamics (Equation (1)) and dynamics (Equation (5)). This model has greatly inspired the researchers of BNBs, as it puts the stability of BNBs in the frame of classical thermodynamics and dynamics. However, for the typical size of BNBs in 100–400 nm, the stability of the BNBs at *ξ* = 0, and how the effect of charge on the nanobubbles is represented are still the problems that cannot be solved by this model. Similarly, Manning [34] proposed a curvature-dependent surface-tension model induced by the Tolman length δ, *γ*(*R*) = *γ*_w_(1 − 2δ/*R*), which could also explain the stability of BNBs thermodynamically. However, besides the above common defects, it is difficult to show the effect of δ~0.2 nm on bubbles of 100 nm.

In addition, Ohgaki et al. [82] and Zhang et al. [83], respectively, proposed the “hard hydrogen bond” structure and super-skin model to interpret the BNBs stability. However, successive experiments have confirmed that the gas inside the BNBs and the external solution are permeable and respond significantly to pressure changes [41,42,43,84,85], and the hard hydrogen bond structure lacks sufficient chemical evidence. Hence, these models are not widely accepted.

## 4. Summary and Outlook

As reviewed above, the stability of BNBs involves many complicated factors, such as charge [27,28], external pressure [41,42,43,84,85,86], temperature [87,88], gas type [54,89], internal density [90], surface contaminants [29,30], experimental detection challenges [3,35,36,37,38], MD challenges [58,59,60], and so on, which has also led to the stability mechanism being difficult to thoroughly solve and being subject to controversy and doubt [2,3,4,5,35,36,37,38,91,92,93,94]. However, looking back at the widely existing practical applications, one will realize that the existence of pre-accepted BNBs may have extraordinary significance for many fields [9,10,11,12,13,14,15,16,17,18,19,20,21,22,23,95,96,97,98,99]. Furthermore, some pioneers have made great efforts to provide us with some valuable exploration. Although the single models mentioned above cannot entirely solve the stability problem of BNBs, one can try to combine two or more models to develop new models.

It is believed that two or more combinations have great potential in the current progress. The first is an “interface charge + contaminant + thermal fluctuation” model, which may solve the major defects of any single model. However, how ions and surfactants are coupled, how the interface layer structure is precisely analyzed, and how thermal fluctuations of the interface layer are regulated are to be further explored.

The second is an “interface charge + contaminant + cluster” model. Experiments and simulations have confirmed that clusters can survive in a locally supersaturated environment [31,32,59,60]. However, how clusters with 100 nm aggregation can survive for a long time may require enrichment of surface-active substances and charges around the aggregates to protect the gases with high chemical potentials inside from being dissolved by diffusion.

These speculations and visions require the development/application of new experimental methods and theoretical models. In particular, molecular simulation techniques (including MD) can provide a useful tool for the analysis of the microstructure of nanobubble systems. The current molecular simulation force field is not enough to fully reveal the real state of BNBs in the experimental environment. Therefore, it is necessary to further develop new force fields, such as machine-learned potentials (MLPs), to simulate larger, more complex, and more realistic nanobubble systems. At the same time, it is necessary to develop new experimental techniques to achieve more accurate detection of the properties of the interior of nanobubbles and their interfaces, such as the internal gas density, interfacial structure, and so on. The current experimental methods, such as DLS, NTA, Zeta potential meter, etc., have certain deficiencies in the detection of BNBs, which are explained in detail in the review by Zhou et al. [3]. In conclusion, at present, the stability studies on BNBs, both MD and experimental, face great challenges and have a long way to go.

One needs to realize that each of the above theoretical models has introduced additional factors in the framework of classical theory. This inspires us to some extent; is there a theory in the classical theory that is no longer applicable in the BNBs system? The two main theories that cause the Laplace catastrophe of bulk nanobubbles are the Laplace equation, Δ*p* = 2*γ*/*R*, and Henry’s law, *p_g_* = *Hc*_gas_. Given the response of nanobubbles to environmental pressure and degassing [41,42,43], here we still adhere to the view that the interior and exterior of the bubble are interconnected; that is, the gases inside and outside the bubbles can be exchanged, so it is assumed that Henry’s law remains valid. Therefore, it is likely that the Laplace equation is no longer applicable to BNBs. This failure may be reflected in the thermal fluctuations of the bubble interface. This is also one of the reasons we believe that the thermal fluctuation model of the interface is a very promising model to explain the stability of BNBs. Furthermore, some relevant simulations and theoretical reports have been performed [70,75]. Thoughtfully, there may be some factors that affect the stability of BNBs that have not yet been revealed, which may affect the applicability of the Laplace equation to BNBs. That is, its macroscopic continuity becomes discrete because of some properties of BNBs extended to the nanoscale. Although there are reports confirming that the Laplace equation is still applicable in a few nanometers [71,72,100], it may not include some special factors of BNBs, such as the specific interface layer.

In reality, BNBs have indeed played increasingly important roles in various application fields, especially in disease diagnosis and therapy in biomedicine, such as ultrasound imaging and drug delivery [2,3,4,5,97]. On the one hand, the stability issue of BNBs is a “dark cloud” over the BNBs field, but on the other hand, it also presents an opportunity due to the possibility of comprising some undiscovered scientific laws. It will be fascinating to explore these unknown laws via BNBs.
